# Potential of miRNAs in Plasma Extracellular Vesicle for the Stratification of Prostate Cancer in a South African Population

**DOI:** 10.3390/cancers15153968

**Published:** 2023-08-04

**Authors:** Dada Oluwaseyi Temilola, Martha Wium, Juliano Paccez, Azola Samkele Salukazana, Hasan H. Otu, Giuseppina M. Carbone, Lisa Kaestner, Stefano Cacciatore, Luiz Fernando Zerbini

**Affiliations:** 1 International Centre for Genetic Engineering and Biotechnology (ICGEB), Cape Town 7925, South Africa; 2 Integrative Biomedical Sciences Division, Faculty of Health Sciences, University of Cape Town, Cape Town 7925, South Africa; 3 Division of Urology, University of Cape Town, Groote Schuur Hospital, Cape Town 7925, South Africa; 4 Department of Electrical and Computer Engineering, University of Nebraska-Lincoln, Lincoln, NE 68588, USA; 5 Institute of Oncology Research (IOR), Università della Svizzera italiana, 6900 Bellinzona, Switzerland

**Keywords:** prostate cancer, extracellular vesicles, exosomes, miRNA, miR-194-5p, miR-16-5p, South Africa

## Abstract

**Simple Summary:**

Prostate cancer (PCa) is the most lethal cancer among African men. Extracellular vesicles (EVs), including exosomes, are released from cancer cells as a form of intercellular communication that can promote cancer growth, increasing invasion and metastasis. EVs are nano-sized vesicles that contain cargo such as microRNA (miRNA), mRNA, and proteins. miRNAs are non-coding RNA that regulate gene expression and are partly responsible for the cancer-promoting function of EVs. As PCa is more aggressive in African populations, it is vital to know which miRNAs are within the EVs of these patients. In this study, we identify and quantify the EV miRNAs in blood plasma from South African patients with low and high Gleason score PCa (an indication of cancer’s aggressive nature). In addition, we use quantitative PCR to evaluate the EV miRNA levels in benign prostatic hyperplasia (BPH) compared to PCa to identify putative biomarkers for the South African population.

**Abstract:**

Prostate cancer (PCa) is the most common cause of cancer death among African men. The analysis of microRNAs (miRNAs) in plasma extracellular vesicles (EVs) can be utilized as a non-invasive tool for the diagnosis of PCa. In this study, we used small RNA sequencing to profile miRNAs cargo in plasma EVs from South African PCa patients. We evaluated the differential expression of miRNAs between low and high Gleason scores in the plasma EVs of South African patients and in the prostatic tissue from data available in the Cancer Genome Atlas (TCGA) Data Portal. We identified 7 miRNAs differently expressed in both EVs and prostatic tissues. We evaluated their expression using qPCR in a larger cohort of 10 patients with benign prostatic hyperplasia (BPH) and 24 patients with PCa. Here, we reported that the ratio between two of these miRNAs (i.e., miR-194-5p/miR-16-5p) showed a higher concentration in PCa compared to BPH and in metastatic PCa compared to localized PCa. We explored for the first time the profiling of miRNAs cargo in plasma EVs as a tool for the identification of putative markers in the South African population. Our finding indicated the ratio miR-194-5p/miR-16-5p as a non-invasive marker for the evaluation of PCa aggressiveness in this population.

## 1. Introduction

Prostate cancer (PCa) is the most prevalent cancer among men worldwide, with more than 375,000 deaths per year [1], and is particularly more aggressive among African men [2]. However, it is unclear whether African patients harbor inherently biologically aggressive diseases, as has been described in migrant populations of African descent, or whether they have simply presented late diagnosis, which may impact disease management. A few studies have been conducted to understand the different genomic [3], proteomic [4,5], and metabolomic [6] profiles in PCa patients with African ancestry.

Diagnosis and monitoring of disease progression are dependent on intrusive procedures like tissue biopsies, which are painful and uncomfortable for the patient. The Gleason score is the most used grading system to determine the aggressiveness of PCa based on the histological appearance of the prostate tissue. Higher grades correspond with the increasing abnormality and dysregulation of cellular processes involved in cancer progression. The transformation from well-differentiated to poorly differentiated cells is indicative of underlying pathophysiological changes that drive the progression of cancer, such as genetic mutation, angiogenesis, hormonal changes, invasion, and metastasis.

Prostate-specific antigen (PSA) is currently used for PCa screening [7], and together Gleason scores to plan the most appropriate course of treatment [8]. Although PSA has a high sensitivity for detecting PCa, its specificity is low [9,10]. Therefore, diagnosis needs to be confirmed with needle biopsies. Additionally, PSA screening also leads to the overdiagnosis of clinically insignificant PCa and the subsequent overtreatment thereof [11]. Subsequently, there is a need for new and better biomarkers for the diagnosis, treatment monitoring, and prognosis of PCa. Although extensive research into prognostic biomarkers is ongoing abroad, it is still being determined whether these will be applicable to the African population. 

Extracellular vesicles (EVs) are nano-sized bilayer lipid vesicles released by almost all cell types, including cancer cells [12,13,14]. EVs include several types of vesicles, such as exosomes (formed in the endosome), microvesicles (formed by the outward budding and fission of the plasma membrane), and apoptotic bodies (released during apoptosis). EVs have been recently applied as a non-invasive tool for patient stratification. EVs play a major role in intercellular communication because they transport protein, DNA, mRNA, miRNA, and lipid molecules between cells [15,16]. EVs contain proteins that function in penetration, invasion, and fusion events, such as tetraspanins (CD9, CD63, CD81, CD82); proteins participate in antigen binding and presentation, such as heat shock proteins (HSP70, HSP90); and including membrane transport and fusion proteins (annexins and Rab) [17]. These proteins enable the uptake of EVs by other cells. Enriched proteins such as TSG101, HSP70, CD81, and CD63 are also commonly used as EV markers [18].

More EVs are released from cancer cells than normal cells, and these EVs contain cancer-related molecules, such as mRNA, miRNA, long non-coding RNA, proteins, and metabolites [19]. Previous studies have investigated EV cargo as biomarkers for the diagnosis and prognosis of different cancer types, including PCa [20,21,22]. Studies have shown blood and urinary EVs from PCa patients possess PCa-specific components, which can serve as biomarkers for the diagnosis of PCa metastasis [23,24]. We previously reported that miR-424-positive EVs are found at a higher frequency in patients with metastatic prostate cancer compared to primary tumors and benign prostatic hyperplasia (BPH) [25]. Our finding showed the role of EV miR-424 in promoting normal prostate epithelial cells to develop stem-like traits and tumor-initiating properties.

Most biomarker discovery studies are conducted among Western populations, and studies in African populations are sparse. In this work, we used small RNA sequencing to identify and quantify the miRNA cargo in plasma EVs from South African PCa patients. We then selected seven miRNAs differentially expressed between low and high Gleason both plasma EVs and prostatic tissue. Finally, we validated the seven miRNAs in a large cohort of 34 patients using qPCR.

## 2. Materials and Methods

### 2.1. Patient Cohort

The ethical approval was received from the Human Research Ethics Committee, Faculty of Health Sciences, University of Cape Town (HREC 454/2012). Informed written consent was obtained from each participant. The study participants were recruited from Groote Schuur and New Somerset Hospitals in Western Cape province, South Africa. Patients scheduled for prostatectomy or Transurethral resection of the prostate (TURP) diagnosed with either BPH or PCa were approached. In this study, we enrolled 24 PCa and 10 BPH patients. We collected about 6 mL of blood in VACUETTE^®^ EDTA tubes (Kremsmünster, Austria) from each participant. Plasma was collected by centrifuging 1000× *g* for 10 min at 4 °C and stored at −80 °C.

### 2.2. Extracellular Vesicle Isolation and Characterization

EVs were isolated from 1 mL of plasma using the Invitrogen Total Exosome Isolation Kit (from plasma) [cat. #4484450, Waltham, MA, USA] following the manufacturer’s guidelines for isolation with Proteinase K treatment. In short, cell debris was removed with two 20 min room temperature centrifugation steps, first at 2000× *g* and then at 10,000× *g*. The plasma was diluted with PBS in a 2:1 ratio, and proteins were digested with 0.05 volumes of Proteinase K for 10 min at 37 °C. Exosome Precipitation Reagent (0.2 volume) was added to the digested sample before incubating the mixture on ice for 30 min. The EVs were collected with centrifugation at 10,000× *g* for 5 min. The EVs were resuspended in 100 μL PBS (137 mM NaCl, 10 mM Phosphate, 2.7 mM KCl; pH 7.4) and stored at −80 °C. We used transmission electron microscopy (TEM) imaging to characterize the morphology of isolated EVs. EVs sample was diluted 1:100 with double deionized water, and 5 μL was applied on a discharged copper grid for one minute. Excess liquid was removed by blotting the copper grid with filter paper and washing twice with 5 μL of double-deionized water. The grid was stained with 5 μL of 2% Uranyl acetate for one minute and viewed on an FEI T20 Transmission Electron Microscope (Hillsborough, OR, USA). Ten photos were taken randomly across the quadrants of the grid. EVs protein was quantified by Bradford reagent (BioRad, Hercules, CA, USA; Cod. 5000006) using BSA as the standard, and equal amounts of proteins were analyzed by SDS-PAGE (12.5% acrylamide, Bio-Rad, Cod. 1610158). Western blot analysis was done as described in our previously published articles using 30 µg of EV proteins [26,27]. The primary antibodies used was anti-CD63 (cat. #ab217345; Abcam, Cambridge, UK), anti-CD9, anti-CD81 (cat. #ab125011; Abcam), and anti-Calnexin (cat. #ab179467; Abcam).

### 2.3. TEM Image Analysis

TEM images saved in TIFF format (grayscale, 2048 × 2048 pixels) were analyzed using the R package EBImage(version 4.42.0). The images were imported into the R environment using the function readImage. After a normalization step, the EVs were identified using the Otsu algorithm [28], and holes were filled using the function fillHull. Overlapped EVs were separated, performing a watershed transformation and watershed-based object detection using the function watershed. The features of shape were quantified for each object in the processed image. Only objects with a ratio standard deviation/mean of the radius below 0.15 were selected. The R code is freely available on GitHub (http://github.com/tkcaccia/EVs-by-TEM, accessed on 15 July 2023).

### 2.4. Small RNA Sequencing in Plasma EVs

Extraction of total RNA was done using 500 μL each of isolated EVs using the Invitrogen Total Exosome RNA & Protein Isolation Kit (cat. #4478545). Extracted RNA was eluted in 50 μL nuclease-free water and stored at −80 °C. Sample quality control for total EV RNA was done using the Agilent Technologies 2100 Bioanalyzer with the High Sensitivity RNA Analysis kit (per manufacturer protocol). The sequencing library preparation was prepared from 10 ng of total EV RNA using the SMARTer smRNA-Seq Kit. Small RNA sequencing was performed on Illumina HiSeq 2500 platform (Illumina, San Diego, CA, USA) with pair-end reads. Sequenced data did each paired and singleton read were concatenated in a single file per library, and overlapping paired-end reads were merged with the Bbmerge from BBMap package (version 39.00) [29]. FASTAQ files were uploaded for annotation using the OASIS web tool and reference genome (*Homo sapiens*—hg38) [30]. Identifiers of mature miRNA were mapped to their stem-loop sequence using the Bioconductor package miRBaseConverter (version 1.12.0). miRNA read counts were normalized for the library size using Trimmed Mean of M-values (TMM) scaling implemented in the function normLibSizes of the Bioconductor package edgeR [31] followed by transformation into log_2_ counts per million.

### 2.5. TCGA Prostatic Tissue miRNA

Tissue miRNA profiling data and the corresponding clinical information were obtained from The Cancer Genome Atlas (TCGA) repository using The Broad Institute Firehose pipeline (http://gdac.broadinstitute.org, accessed on 10 July 2023). Primary samples from the prostate adenocarcinoma (PRAD) dataset were inferred using the TCGA sample code “01A”, which is the two-digit code following the TCGA legacy sample name (limiting the analysis to a sample for each patient).

### 2.6. MicroRNA Enrichment

The MIENTURNET (microRNA Enrichment TURned NETwork) web tool [32] was used to perform miRNA-target enrichment analysis and regulatory network in order to investigate genes targeted by the miRNAs. The regulatory network includes strong and weak interactions between miRNAs and their targets, as defined by Licursi et al. [32]. Strong interactions are interactions validated by “strong” experimental evidence (e.g., Luciferase assay, Western); weak interactions consider weaker experimental evidence (e.g., CLIP). Functional enrichment analysis of miRNA targets was obtained using the WikiPathways gene set [33]. The over-represented analysis was done using miEAA2.0 [34]. The cellular location of the miRNAs was predicted using miRNALoc [35], and FANTOM5 mammalian expression miRNA atlas [36] was used to predict the cellular origin. The immunological features of TCGA-PRAD prostatic tissue obtained using CIBERSORT [37] and pathological tissue analysis were retrieved from [38].

### 2.7. Reverse Transcription-Quantitative Polymerase Chain Reaction (RT-qPCR)

We performed qPCR to evaluate the expression levels in a larger cohort from 26 Pca and 10 BPH EV RNA samples. Complementary DNA (cDNA) was prepared using Qiagen miRCURY LNA RT Kit (Hilden, Germany; cat. #339340) following the manufacturer’s protocol. This was followed by a real-time PCR expression analysis of the miRNAs identified at the initial experimental phase. The miRCURY LNA miRNA PCR Assay used are commercially available from Qiagen: (hsa-miR-16-5p, stock code: QIA/339306_YP00205702; hsa-miR-10a-5p, Stock code: QIA/339306_YP00204778; hsa-miR-194-5p, stock code: QIA/339306_YP00204080; hsa-miR-144-5p stock code: QIA/339306_YP00204670; hsa-miR-93-5p, stock code: QIA/339306_YP00204715; hsa-miR-326, stock code: QIA/339306_YP00204512; hsa-miR-221-3p, stock code: QIA/339306_YP00204532; hsa-miR-21-5p, stock code: QIA/339306_YP00204230). Real-time PCR was performed on the LightCycler480 system (Roche Diagnostics, Mannheim, Germany) using the absolute quantification method. The absolute copy number of cDNA was calculated using the standard curve prepared with qPCR experiments.

### 2.8. Statistical Analysis

The negative binomial differential expression method edgeR was used to identify differentially expressed genes [31]. Differences in numerical covariates were evaluated using Wilcoxon and Kruskal–Wallis rank-sum test. Differences between categorical variables (e.g., ethnicity) were assessed using Fisher’s exact test. The correlation coefficient (rho) between miRNA concentrations and other biological features (e.g., genes and immunological features) was calculated using Spearman’s rank test. *p* values were adjusted for multiple testing with the Benjamini–Hochberg correction, and a false discovery rate (FDR) cutoff of 0.1 was used. The statistical analysis was facilitated using the KODAMA R package [39]. Receiver operating characteristic (ROC) curve analysis to evaluate the sensitivity of biomarkers was performed using the pROC R package. 

## 3. Results

### 3.1. Sequencing of EV miRNA from Prostate Cancer Samples

**Figure 1:**
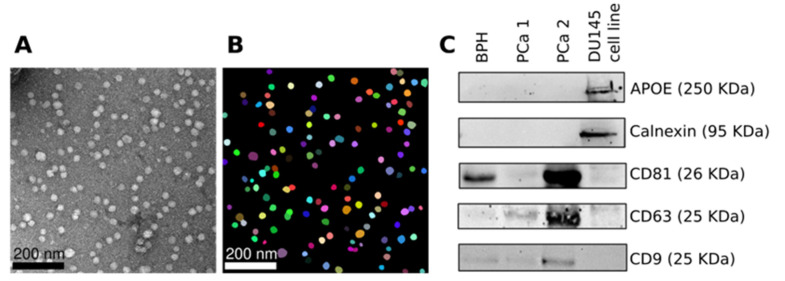
Characterization of EVs derived from patient plasma. (**A**) TEM image of EVs from a representative PCa. EV samples were diluted 1:100, and the imaging was performed with the FEI T20 transmission electron microscope. (**B**) Digital analysis of a TEM image to profile the morphology of EVs. (**C**) Western blot analysis of the EV-positive and -negative markers. The EV-positive markers, CD9, CD63, and CD81, and the EV-negative markers, calnexin, and APOE, were evaluated by immunoblotting using specific antibodies. We used 30 µg of protein per sample. The total cell lysates from a DU145 cell line culture were used as a control. PCa, Prostate cancer; BPH, Benign prostatic hyperplasia; TEM, transmission electron microscopy; EV, Extracellular vesicles. See Supplementary Material File S1 for the original image of the Western Blot.

EVs from a cohort of South African patients with PCa were isolated from plasma samples. The isolation EV protocol was validated using multiple techniques [25]. TEM imaging indicated that the isolated EV morphology is consistent with small EVs (Figure 1A). Further image analysis of TEM images (Figure 1B) reveals that more than 90% of EVs have a diameter ranging between 15 nm and 29 nm. We found no statistically significant changes in the dimension of EVs between BPH and PCa. Western blot analysis confirmed the expression of EV-positive markers CD9, CD63, and CD81 and the absence of EV-negative markers ApoE and calnexin (Figure 1C). 

To have insights into the miRNA expression profile, we sequenced the EV miRNA from 3 patients with low Gleason scores (<8) and 3 patients with high Gleason scores (≥8). The clinical data of these patients are shown in Table 1.

A total of 868 miRNAs were identified, of which 298 have average read counts above 10. The differential analysis between low and high Gleason scores identified 65 miRNAs as statistically significantly different (Supplementary Table S1). Using literature searches, we identified 49 miRNAs that have been previously associated with African ancestry in PCa (Supplementary Table S2). None of these overlaps with the 65 deregulated miRNAs identified by this study. This emphasizes the uniqueness of the population and the urgent need for studies like the current one in African populations.

**Figure 2:**
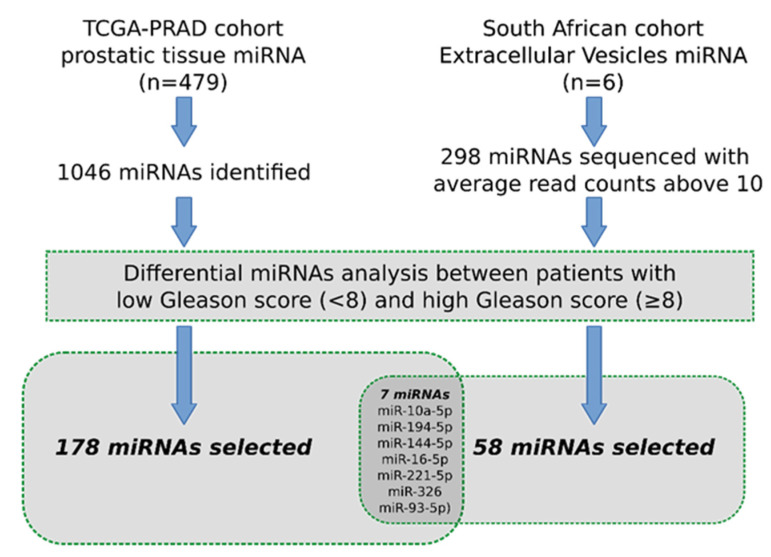
Outline of the differential expression analysis. Differential expression was assessed between low and high Gleason groups in the TCGA prostate cancer tissue miRNA database and extracellular vesicle miRNA sequencing data from the South African cohort. Venn diagram indicated the shared miRNA between the two analyses. TCGA-PRAD, the Cancer Genome Atlas Prostate Adenocarcinoma.

EVs have been implicated in the intercellular transfer of miRNA into extracellular space. EVs are secreted by almost all cell types; in plasma, EVs are mainly derived from blood cells, such as platelets, T-cells, and B cells [40]. In order to identify deregulated miRNA possibly originating in the prostatic cancer tissue, we retrieved miRNA expression profiles of 479 prostatic tumor tissue from data available in the TCGA-PRAD dataset, including 284 with low Gleason scores (<8) and 195 with high Gleason scores (≥8). A total of 185 miRNAs were found deregulated in tissues with higher Gleason scores (Supplementary Table S3). Only seven miRNAs were similarly deregulated in both cohorts (Table 2). A summary of the approach employed in this study is demonstrated in Figure 2.

### 3.2. Identification of EV miRNA Target Genes

We used MIENTURNET’s network analysis [32] to assess the relationships between miRNAs and target genes. miRNA target interaction network and functional enrichment analysis were conducted to assess the biological relevance. We reported 364 candidate targeted genes (*p*-value < 0.05; FDR < 0.1) for the seven previously identified miRNAs (Supplementary Table S4).

**Figure 3:**
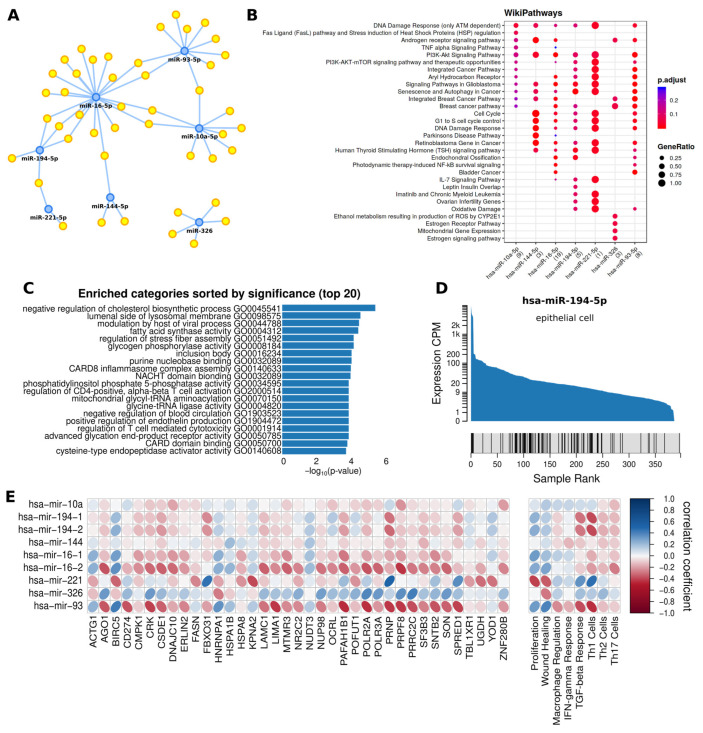
(**A**) Regulatory network of PCa-associated genes and their regulating miRNAs. Blue nodes represent the selected miRNAs. Yellow nodes represent miRNA-associated genes. (**B**) WikiPathways enrichment analysis for the genes with a strong interaction with the selected miRNAs. (**C**) miEAA2.0 enrichment analysis of the selected miRNA. (**D**) FANTOM5 enrichment score plot of miR-194-5p for epithelial cells. (**E**) Correlation map between the selected miRNA and target gene with strong and weak interaction and immunological tumor features.

A network of experimentally validated miRNA–target strong interactions was constructed from the seven selected miRNAs (Figure 3A). Three miRNAs, miR-16-5p, miR-93-5p, and miR-10a-5p, were characterized by a high number of interactions (1557, 1220, and 463, respectively). miR-326, miR-221-5p, miR-194-5p, and miR-144-5p showed respectively 144, 138, 93, and 36 interactions. All strong and weak interactions are reported in Supplementary Table S5. The functional enrichment analysis of the Wiki pathways for the seven is reported in Figure 3B, highlighting a modulation of the androgen receptor (AR) signaling, PI3k-Akt signaling, and inflammation-related pathways (e.g., Il-7 and TNF-alpha signaling). Although not highlighted, AR signaling has also been recently associated with both miR-194-5p [41] and miR-221-5p [42].

The miEAA2.0 analysis [34] yielded 397 enriched subcategories for the seven identified miRNAs (Supplementary Table S6). Figure 3C summarizes the first 20 most significantly enriched subcategories for the seven miRNAs. From this analysis, we reported a possible modulation of Caspase recruitment domain (CARD) signaling and T-cell activation. The analysis corroborated the previous findings that inflammatory pathways are targeted by these miRNAs. 

We also identified that pathways related to the metabolism of lipids (e.g., cholesterol and fatty acids) are also targeted by these miRNAs. Fatty Acid Synthase (FASN), a key enzyme in the de novo synthesis of fatty acids [43] and the lipogenic phenotype of PCa [44,45], was indeed targeted by four out of seven identified miRNAs (i.e., miR-326, miR-10a-5p, miR-93-5p, miR-16-5p). 

FANTOM5 mammalian expression miRNA atlas [36] was used to predict the cellular origin of the seven identified miRNAs (Supplementary Table S7). Leukocyte, neutrophil, myeloid and hematopoietic cells were the putative primary source of miR-144-5p, miR-16-5p, miR-221-5p, and miR-326. The analysis suggested that miR-194-5p, miR-10a-5p, and miR-93-5p were of epithelial origin and may be promising candidates for future investigation as prostatic epithelial tissue-related miRNAs. The highest association with epithelial cells was reported for miR-194-5p (Figure 3D).

The miRNA locations were predicted using miRNALoc [35]. Most of the miRNAs were predicted as expected to be localized in exosomes, including miR-10a-5p, miR-194-5p, miR-144-5p, and miR-326. miR-93-5p and miR-16-5p were predicted to be localized in extracellular vesicle and miR-221-5p in microvescicle (Supplementary Table S8). Gene expression data from the prostatic cancer cohort in TCGA was used to investigate the correlation between the targeted gene and miRNA (Figure 3E). The correlation between miRNA and immunological features was also analyzed (Figure 3E), reporting a strong negative correlation between the Th1 cell population and the miR-194-5p precursors, miR-194-1 and miR-194-2.

### 3.3. Validation of miRNA Expression by RT-qPCR Analysis

To validate our findings, we quantified the expression of the seven miRNAs in a larger cohort of 24 PCa and 10 BPH patients. The latter was included as a control in order to identify changes cancer-specific comparing a benign condition of the prostate with a malignant one. The clinical data from all patients are summarized in Table 3. Additional details are provided in Supplementary Table S9. 

**Figure 4:**
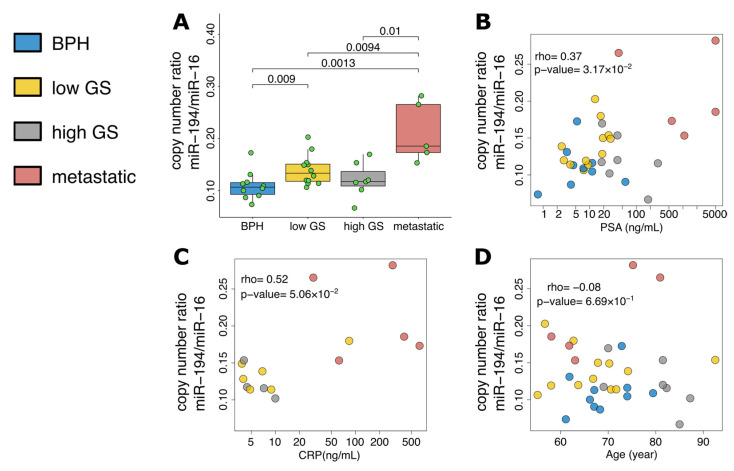
(**A**) Box-whiskers plot of the ratio miR-194-5p/miR-16-5p and severity group. Correlations between the ratio miR-194-5p/miR-16-5p and (**B**) PSA, (**C**) CRP, and (**D**) Age.

**Figure 5:**
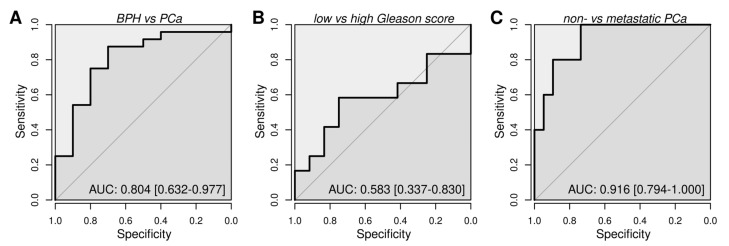
ROC curve of the ratio miR-194-5p/miR-16-5p analyzing (**A**) BPH and PCa patients, (**B**) PCa patients with low and high Gleason Scores, (**C**) PCa without and with metastatic disease.

The analysis between the severity of PCa and absolute values of extracellular vesicle miRNA did not show a clear trend (Supplementary Figure S1). Ratios between miRNA pairs were calculated (Supplementary Table S10). Using miR-16-5p as an endogenous reference gene for the normalization of extracellular vesicles miRNA expression, miR-194-5p showed the highest correlation with the severity of PCa (rho = 43.3; *p*-value = 0.00955) in Figure 4A. A strong correlation was also reported for PSA (Figure 4B) and CRP (Figure 4C). The sample size of this cohort is limited, and no statistically significant change was reported for the age (Figure 4D). In addition, a lower miR-194-5p value was observed in the Black patients with PCa (*p*-value < 0.05). ROC curves (Figure 5) showed a good prediction of the ratio miR-194-5p/miR-16-5p when comparing BPH with PCa (Figure 5A) and non-metastatic with metastatic PCa (Figure 5C). 

## 4. Discussion

Following FDA breakthrough device designation approval of the first EV cargo diagnostic test, ExoDx Prostate Intelliscore (EPI), EV markers are increasingly being explored for their diagnostic and prognostic potential [46]. For PCa, both urinary and blood EV cargo are exploited for their prostate-cancer-specific contents [23,24]. Noticeably miRNA cargo was found to be promising putative diagnostic and prognostic markers for PCa [47]. This study aimed to explore EV miRNA cargo in the South African PCa population. We isolated the EVs from the plasma and assessed their morphological characterization with TEM image analysis. Although the majority of EVs isolated by our protocol range between 15 nm and 29 nm, a quantitative technique, such as Dynamic Light Scattering, may improve the EV size characterization. 

We identified 65 miRNAs deregulated in EVs of PCa patients with high Gleason scores that have not previously been reported to be associated with PCa in patients with African ancestry. Some of them were associated with other oncological diseases, which emphasizes the unique contribution of our study to PCa literature on the African population. In this study, we compared our plasma EV miRNA sequenced data and prostate tissue miRNA data in the TCGA database. We found seven differentially expressed miRNAs in both EVs and prostate tissue. It is well-known that around 90% of patients recruited in the TCGA have a predominant European ancestry. The selection of shared deregulated miRNAs in our and TCGA cohorts may have led to excluding miRNAs deregulated only in the South African population. On the other hand, this approach may lead to the identification of putative biomarkers that show high accuracy in more heterogeneous populations.

The role of AR receptor in PCa progression is well-establish [48]. All seven miRNAs were associated with AR signaling. Growing evidence indicates that the tumor microenvironment contributes to antiandrogen resistance [49]. Considering that the putative primary source for four of the seven identified miRNAs is immune cells, we speculate on the tumor microenvironment’s possible role in regulating the AR signaling through EVs released by non-epithelial cells. The deregulation of AR signaling could, in part, justify the link with the other pathways identified as targets. Androgens, mediated by the AR, stimulate the expression and activity of FASN [50] and are linked to a rearrangement of the metabolic pathways [44].

We assessed the miRNA expression in EVs isolated from the blood of additional patients to validate the miRNAs identified by sequencing. We found that the miR-194-5p/miR-16-5p expression ratio correlates with the disease severity to serve as potential biomarkers for PCa diagnosis. The miR-194-5p/miR-16-5p ratio significantly separates BPH samples from low Gleason score PCa samples and metastatic PCa from low and high Gleason score PCa. 

According to previous research, miR-194 is a good candidate marker for high-risk and metastatic prostate cancer [51,52,53], which suggests our experimental approach was valid and robust. The literature reports that higher levels of miR-194 regulate cancer by increasing migration, invasion, and epithelial-mesenchymal transition [51,52]. In cell lines, ectopic delivery of miR-194 results in increased migration, invasion, and epithelial–mesenchymal transition. Stable miR-194 overexpression led to metastasis of intravenous and intraprostatic tumor xenografts [51]. Localized PCa patients have significantly lower serum levels of miR-194 compared with metastatic castrate-resistant prostate patients [54]. Additionally, miR-194 is a driver for metastasis in PCa [51] and is associated with poor outcomes. It is also a marker for biochemical recurrence following radical prostatectomy [54]. 

miR-16 has been used as an endogenous marker to normalize qPCR results in many studies [55,56,57,58,59,60]. Normalization is required as the EV concentration in the plasma of patients differs. Many research papers report an increase in the concentration of plasma EVs in cancer patients compared to healthy people and with the disease severity [61,62,63,64]. This could possibly explain why the ratio of miR-194-5p/miR-16 was better in distinguishing BPH from PCa patients than the single miRNAs. However, other authors report that miR-16-5p is associated with PCa disease severity [65,66]. It was shown that miR-16-5p is upregulated in PCa patients and that this is associated significantly with high-risk Gleason scores and with PSA levels [65]. Increased levels of miR-16-5p were reported in advanced PCa compared to localized PCa and BPH. Additionally, miR-16-5p was incorporated into a model along with three other miRNAs (miR-375, miR-33a-5p, and miR-409-3p) and PSA that can predict the outcome of transrectal ultrasound-guided biopsies better that PSA alone [66]. 

Regarding racial disparities, it was reported that miR-194 has higher expression levels in Nigerian women with breast cancer than in the British Caucasian, British Black, and Indian groups [67]. A systematic review and meta-analysis of the miR-16 family found that ethnicity may influence miRNA concentration; unfortunately, data from African patients were unavailable, and only Asian and Caucasian groups were assessed [68]. Our study observed lower miR-194-5p levels in the Black ethnic group. This emphasizes the importance of our research, as the genetic composition of populations will influence the robustness of biomarkers. Unfortunately, African populations are frequently ignored in research even though Africa has the lion’s share of PCa deaths. 

## 5. Conclusions

Studies have demonstrated the correlation between miR-194 and metastasis, poor outcomes, and recurrence, and miR-16 as an endogenous marker used to normalize qPCR results in PCa. However, the combination of these two miRNAs as potential biomarkers for PCa, most notably in an African population, is new. Our study expands the understanding of the potential of EV miRNA cargo as diagnostic and prognostic markers for PCa. The identification of specific miRNAs, such as miR-194-5p/miR-16-5p ratio, provides valuable insights for developing improved diagnostic tools and personalized treatment strategies for PCa patients. Furthermore, our research highlights the importance of inclusive research that considers diverse populations, including African populations, to ensure the effectiveness and applicability of biomarkers across different genetic backgrounds.

## Figures and Tables

**Table 1:** Clinical data from Prostate cancer patients used for miRNA profiling.

Samples	Age (Year)	PSA (ng/mL)	Ethnicity	Gleason Score	Clinical Stage	NCCN Classification
SAPC0164	74.2	2.5	Black	3 + 3	T1a	Very low
SAPC0203	66.8	18.6	Coloured	3 + 3	cT2a	Intermediate
SAPC0238	92.5	24.4	Coloured	3 + 3	T1a	High
SAPC0185	69.0	18.0	Coloured	4 + 5	T2c	High
SAPC0195	87.2	26.5	Black	4 + 5	T3	Very high
SAPC0180	58.1	>5000	Black	5 + 5	T3/T4	Very high

PSA, Prostate-Specific Antigen; NCCN, National Comprehensive Cancer Network.

**Table 2:** Significant deregulated miRNA commonly expressed in the same direction between TCGA miRNA and EV miRNA.

		EV miRNA		TCGA PCa Tissue miRNA		
	**LogFC**	***p*** **-Value**	**FDR**	**LogFC**	***p*** **-Value**	**FDR**
hsa-miR-10a-5p	9.21	2.78 × 10^−4^	2.92 × 10^−3^	0.30	9.52 × 10^−5^	1.11 × 10^−3^
hsa-miR-194-5p	3.45	5.11 × 10^−3^	3.34 × 10^−2^	0.25	3.41 × 10^−6^	5.95 × 10^−5^
hsa-miR-144-5p	2.30	5.72 × 10^−3^	3.56 × 10^−2^	0.27	7.70 × 10^−3^	4.91 × 10^−2^
hsa-miR-16-5p	1.89	1.67 × 10^−2^	8.05 × 10^−2^	0.19	4.42 × 10^−6^	7.34 × 10^−5^
hsa-miR-221-5p	−2.08	1.83 × 10^−2^	8.51 × 10^−2^	−0.55	1.55 × 10^−12^	6.77 × 10^−11^
hsa-miR-326	−2.15	1.84 × 10^−2^	8.51 × 10^−2^	−0.33	5.26 × 10^−4^	4.83 × 10^−3^
hsa-miR-93-5p	1.67	1.85 × 10^−2^	8.51 × 10^−2^	0.35	9.74 × 10^−9^	2.55 × 10^−7^

EV, Extracellular vesicle; FDR, False Discovery Rate; LogFC, Log fold change; PCa, prostate cancer; TCGA, The Cancer Genome Atlas.

**Table 3:** Clinical data from patients used for qPCR.

Feature	BPH (n = 10)	Low GS (n = 12)	High GS (n = 7)	Metastatic (n = 5)	*p*-Value
Age (year), median [IQR]	68 (66–74)	67 (62–71)	81 (76–84)	63 (62–75)	3.66 × 10^−2^
PSA (ng/mL), median [IQR]	5.3 (3.9–11.3)	11.1 (6.5–18.7)	39.3 (22.5–109)	1070 (576–5000)	2.25 × 10^−4^
Ethnicity					1.50 × 10^−1^
Black, n (%)	6 (60.0)	1 (8.3)	3 (42.9)	2 (40.0)	
Coloured, n (%)	4 (40.0)	9 (75.0)	3 (42.9)	2 (40.0)	
White, n (%)	0 (0.0)	2 (16.7)	1 (14.3)	1 (20.0)	
CRP (ng/mL), median [IQR]	-	5.9 (4.2–8.4)	5.8 (4.3–7.9)	291 (62–405)	1.89 × 10^−2^

IQR, interquartile range; BPH, benign prostatic hyperplasia; GS, Gleason Score; CRP, C-reactive protein; PSA, prostatic specific antigen.

## Data Availability

All data generated and analyzed during the current study are available in the Supplementary Table file. Any remaining data can be obtained from the corresponding author upon reasonable request.

## Supplementary Materials

The following supporting information can be downloaded at: https://www.mdpi.com/article/10.3390/cancers15153968/s1, Supplementary Table file, containing: Supplementary Table S1: Differential expressed EV miRNAs in low Gleason compared to the high Gleason groups in the South African prostate cancer patients; Supplementary Table S2: miRNAs reported in the literature to associated with African ancestry in prostate cancer; Supplementary Table S3: Differential expressed EV miRNAs in low Gleason compared to the high Gleason groups in the TCGA Dataset; Supplementary Table S4: Mienturnet Enrichment results with miRTarBase using the seven deregulated miRNAs as input; Supplementary Table S5: The strong and weak interactions in the MiRNA-target genes network analysis (miRTarBase) using the seven miRNA set as input; Supplementary Table S6: The gene ontology results predicted the miEAA 2.0—miRNA Enrichment and Annotation tool using the seven miRNA set as input; Supplementary Table S7: Cell ontology predictions of the seven miRNA; Supplementary Table S8: Cellular location prediction using miRNALoc tool with the seven miRNA set; Supplementary Table S9: Demographics and clinical data of the South African cohort and copy number of EV miRNA quantified by qPCR; Supplementary Table S10: The correlation between the miRNA pairs and severity of PCa. Figure S1. Box-whiskers plot of the copy number of the seven miRNAs and severity group. File S1: The original image of the Western Blot. References [69,70,71,72,73,74,75,76,77,78,79,80,81,82,83,84] are cited in the supplementary materials.
