# Supplementary figures and images for: Potential of miRNAs in Plasma Extracellular Vesicle for the Stratification of Prostate Cancer in a South African Population
Source: Cancers (Basel). 2023 Aug 4;15(15):3968. doi: 10.3390/cancers15153968 (PMC10417259; doi:10.3390/cancers15153968)

**Figure S1.** Box-whiskers plot of the copy number of the seven miRNAs and severity group.

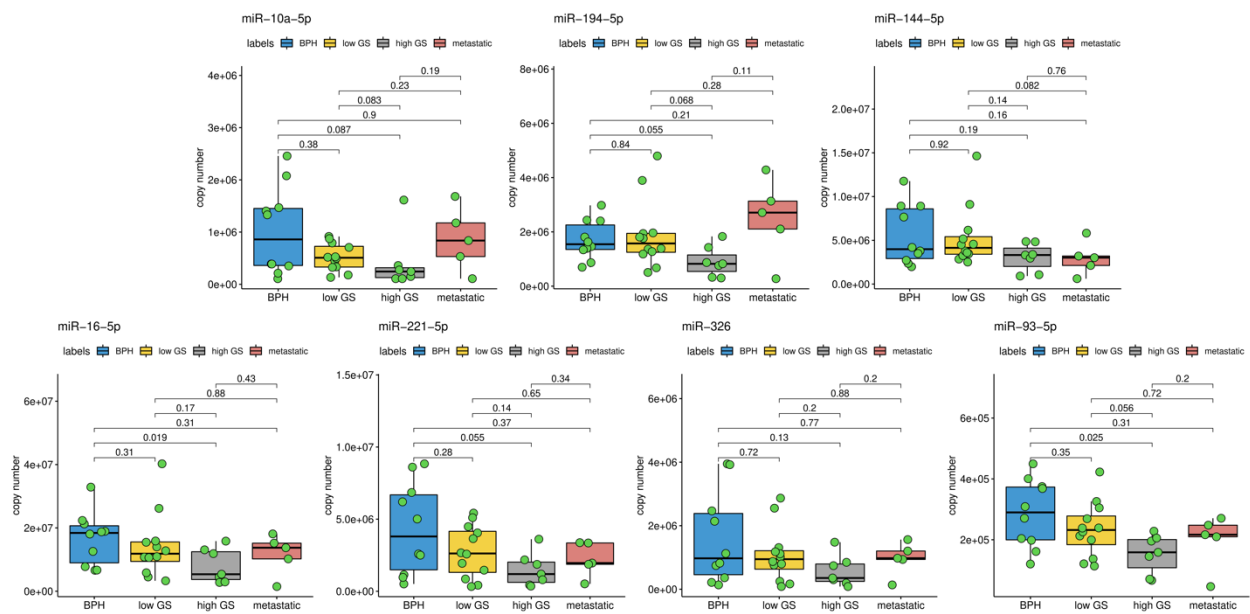

Supplement: Supplementary file 1 [file cancers-15-03968-s001.zip › Supplementary Figure S1.pdf]

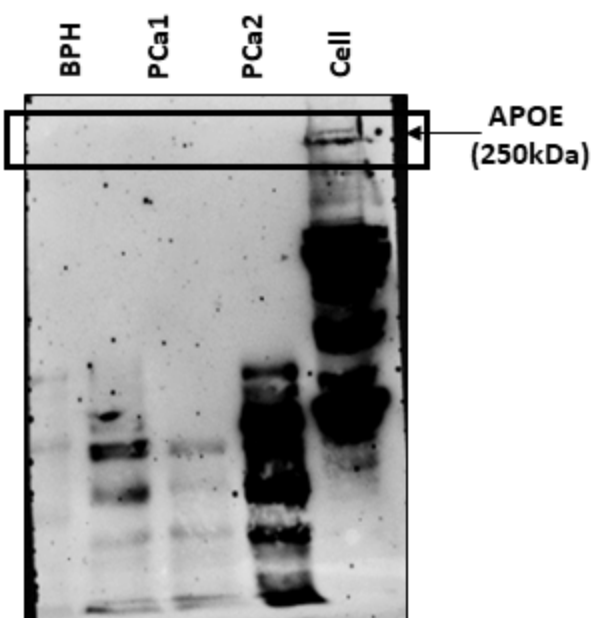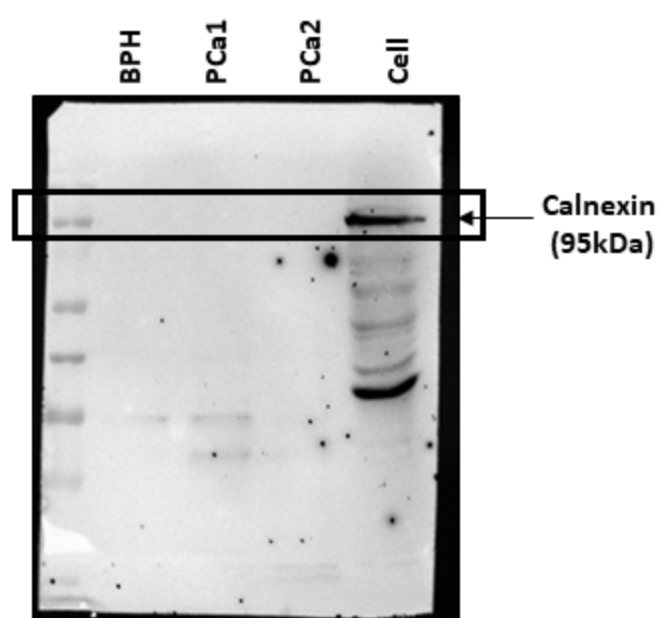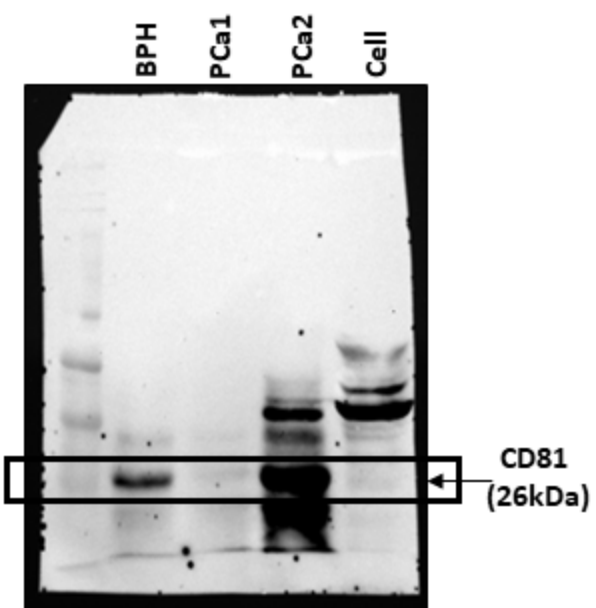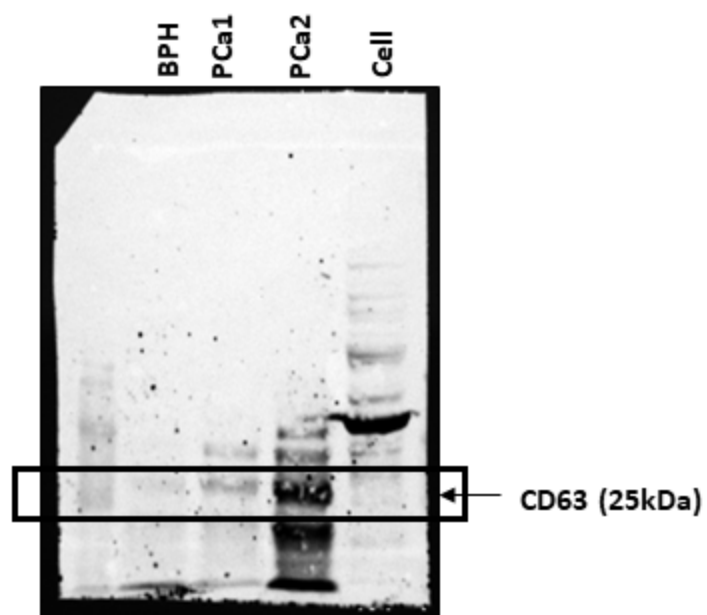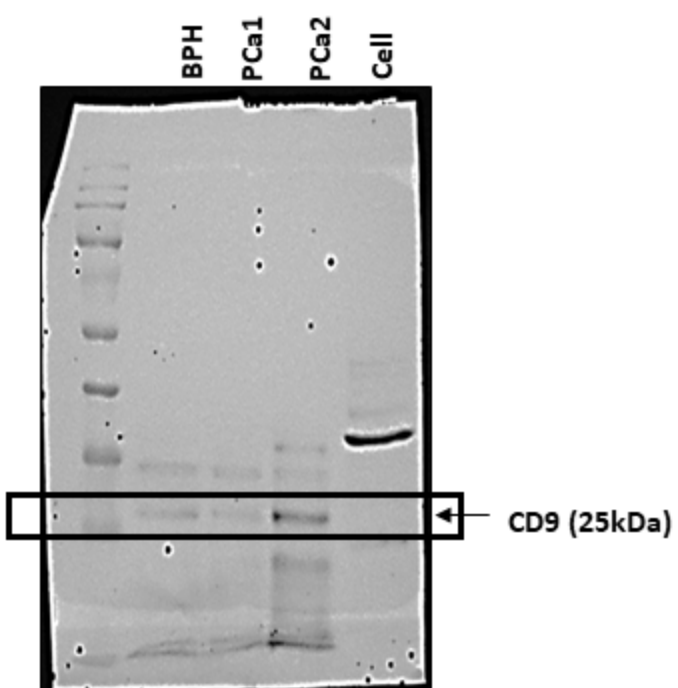

Supplement: Supplementary file 1 [file cancers-15-03968-s001.zip › Supplementary Material File S1.pdf]
